# *Mycobacterium tuberculosis* SIT42 Infection in an Abused Dog in Southern Italy

**DOI:** 10.3389/fvets.2021.653360

**Published:** 2021-06-22

**Authors:** Lucia Vangone, Lorena Cardillo, Marita Georgia Riccardi, Giorgia Borriello, Anna Cerrone, Paolo Coppa, Roberto Scialla, Emanuela Sannino, Gianluca Miletti, Giorgio Galiero, Giovanna Fusco

**Affiliations:** ^1^Department of Animal Health, Unit of Forensic Veterinary Medicine and Anatomopathology, Istituto Zooprofilattico Sperimentale del Mezzogiorno, Naples, Italy; ^2^Department of Animal Health, Unit of Virology, Istituto Zooprofilattico Sperimentale del Mezzogiorno, Naples, Italy; ^3^Department of Animal Health, Unit of Applied Biotechnologies and Bioinformatics, Istituto Zooprofilattico Sperimentale del Mezzogiorno, Naples, Italy; ^4^Department of Animal Health, Unit of Special Diagnostics and Fish Pathology, Istituto Zooprofilattico Sperimentale del Mezzogiorno, Naples, Italy; ^5^Istituto Zooprofilattico Sperimentale del Mezzogiorno, Naples, Italy

**Keywords:** canine, tuberculosis, *Mycobacterium tuberculosis*, SIT42, necropsy, abuse

## Abstract

A case of *Mycobacterium tuberculosis* infection is described in a dead adult male dog in Southern Italy. The carcass was found by the Health Authority in a gypsy encampment. It was admitted to our forensic veterinary medicine unit, with a suspicion of cruelty to the animal. Necropsy showed beating and traumatism signs, and mistreating was confirmed. Gross lesions included multiple nodular hepatic lesions, hemorrhagic enteritis with enlarged mesenteric lymph nodes, body cavity effusions, and an adrenal neoplasm. Bacteriological and molecular analyses were carried out on the liver lesions that enabled to identify *M. tuberculosis* SIT42 (LAM9). Drug-resistance patterns were evaluated by screening mutations on the *rpoB* and *katG* genes that showed susceptibility to both rifampin and isoniazid, respectively. Very few studies report canine tuberculosis, and little is known about the disease in Italy. To the authors' knowledge, this is the first report of *Mycobacterium tuberculosis* SIT42 infection in a dog in Italy.

## Introduction

*Mycobacterium tuberculosis* (*Mtb*) is one of the causative agents of tuberculosis (TB), a chronic disease that affects both animals and humans. Around 10 million new human cases are diagnosed every year, and it is classified as 1 of the top 10 causes of death worldwide (1). A few studies have reported TB incidence in dogs, and most cases have identified *M. tuberculosis* as the prevalent species (2, 3), although cases of *M. bovis, M. avium, M. pinnipedi, M. africanum*, and *M. microti* infections have been occasionally described (4–10).

A close contact with infected humans or other susceptible animal species represents the main risk factor for dogs; thus, it has been speculated that humans could represent a source of infection for dogs (4, 11, 12). On the other hand, Martinho et al. (13) observed that, in case of generalised infection, dogs can shed *Mtb* by nasal secretion, urine, and faeces, causing environmental contamination, so that they could act a role in the transmission of the pathogen to humans and other animal species (4).

In naturally infected dogs, TB commonly shows a subclinical course for long periods (9, 14), but, when clinical signs are developed, they are described to affect primarily lungs and regional lymph nodes; nevertheless, different localizations have been described (15, 16). The ante mortem diagnostic approach of canine TB is difficult for the absence of symptoms in infected dogs (9, 14), complicated by the absence of a validated immunological assay (3, 12). Indeed, this species shows poor response to tuberculin skin test (TST) injection both of *M. tuberculosis* (Manteaux test) and *M. bovis* purified protein derivate (PPD) tuberculin and a high percentage of false negatives and false positives to serological examinations, too (12, 14, 17).

## Methods

### Anatomopathological and Microbiological Examinations

The dog was submitted to a complete necropsy performed by post-graduated veterinarians of the Unit of Forensic Veterinary Medicine according to standard protocols (18). Suspected lesions were collected and submitted to histopathological analysis, stained with hematoxylin and eosin (H&E) and microbiological examinations.

Notably, *M. tuberculosis* was determined according to the procedures described by the World Organisation for Animal Health in the Manual of Diagnostic Tests and Vaccines for Terrestrial Animals (19). Samples from liver were homogenised and decontaminated. The bacteriological culture was first performed by using the BD BACTEC Mycobacteria Growth Indicator Tube (MGIT) 960 System (Becton, Dickinson and Company, Franklin Lakes, NJ, USA) according to the manufacturer's instructions and incubated up to 42 days. Therefore, 0.2 ml of liquid media was transferred to the solid Stonebrink TB medium (Becton, Dickinson and Company) and incubated at 37 ± 1°C. The development of characteristic colonies was verified in the first reading after 2–5 days and then weekly for a maximum of 8 weeks starting from the date of inoculation into the liquid medium. Colonies suspected to be *Mycobacterium* spp. were subjected to Ziehl–Neelsen staining.

### Molecular Characterisation

Suspected colonies were subjected to molecular analysis for species identification. First, a real-time PCR targeting a 209-bp fragment of the insertion sequence IS6110 common to all the members of the *Mycobacterium tuberculosis Complex* (*MtbC*) was performed according to the protocol of Chimara et al. (20). For this purpose, 250 μl of a bacterial colony suspension was boiled at 99°C for 15 min and then centrifuged at 10,621 × g for 10 min, and the supernatant was used for molecular analyses. Amplification was carried out in a final volume of 25 μl with the QuantiFast Pathogen kit (QIAGEN, Hilden, Germany) and the following set of primers and probe: forward primer EXT-1 5′-CCCGGACAGGCCGAGTTT-3′ 0.5 μM, reverse primer, INT-1 5′-CCCCATCGACCTACTACG-3′ 0.5 μM, probe IS6110 5′-FAM-AACTCAAGGAGCAGTCAGGCH-BHQ1-3′ 0.2 μM. Thermal cycling conditions included an initial denaturation step at 95°C for 5 min, followed by 45 cycles at 95°C for 15 s and 60°C for 30 s, and were performed on a CFX 96 touch thermal cycler (BIORAD, Hercules, CA, USA). Species identification was performed by the gyrB-restriction fragment length polymorphism (gyrB-RFLP) analysis. For this assay, 5 μl of bacterial DNA was amplified in a final volume of 25 μl including KAPA2G robust HotStart ready mix (Roche) 1X, Primer forward gyrB-Dir 5′ TCGGACGCGTATGCGATATC 3′ 1 μM, and Primer reverse gyrB-Rev 5′ GCGGTTCGCTGACCTTCACCGAGATCAC 3′ 1 μM. PCR amplification was performed on a T-100 thermal cycler (BIORAD) and consisted of one step at 95°C for 5 min followed by 40 cycles at 95°C for 30 s, 60°C for 30 s, and 72°C for 1 min, and a last extension step at 72°C for 10 min. The presence of the PCR product, a fragment of 765 bp, was visualised on the automated capillary electrophoresis system QIAXcell (QIAGEN). Following electrophoresis, the amplicon was digested with 6U of the RsaI enzyme (PROMEGA) at 37°C for 2 h. Restriction fragments were resolved by the QIAXcell system. The species Tuberculosis was identified by the presence of a fragment of 560 bp. All the reactions included negative and positive controls, represented by water and DNA extracted from a reference strain of *M. tuberculosis* and *M. bovis*, respectively. Next, the specimens were submitted to MIRU-VNTR by the National Reference Laboratories for Tuberculosis [Istituto Zooprofilattico Sperimentale of Lombardia and Emilia Romagna (IZSLER), Brescia, Italy] that kindly provided the results.

### Detection of Rifampin and Isoniazid Resistance by DNA Sequencing

Bacterial DNA was analysed for the detection of mutations in the *rpoB* and *katG* genes. PCR amplification was performed using primers katG-F (5′-AGCTCGTATGGCACCGGAAC-3′), katG-R (5′-TTGACCTCCCACCCGACTTG-3′) and rpoB TR1 (5′-TACGGTCGGCGAGCTGATCC-3′), rpoB3-R (5′-GTACGGCGTTTCGATGAACCCGAA-3′) according to Takawira and colleagues (21) with slight modifications. For each gene, PCR was carried out in a 25-μl reaction mixture containing 5 μl of DNA, 2.5 μl of 10 μM of each primer, 12.5 μl of KAPA2G Robust HotStart (KAPA BIOSYSTEMS, Salt River, Cape Town, South Africa), and 2.5 μl of DNAse/RNAse free water. PCR conditions were as follows: an initial denaturation step at 95°C for 5 min followed by 34 cycles consisting of denaturation at 94°C for 1 min, annealing at 64°C for 30 s, and extension at 72°C for 30 s and final extension at 72°C for 5 min. The DNA of *Mycobacterium tuberculosis* was used as a positive control. The presence of specific amplification products of *rpoB* (412 bp) and *katG* genes (620 bp) was evaluated by automated capillary electrophoresis with QIAxcel Advanced (QIAGEN). The PCR products were purified on silicagel membranes using the QIAquick PCR quantification kit according to the manufacturer's instructions (Qiagen) and bidirectionally PCR sequenced with BigDye Terminator v1.1 Cycle Sequencing Kit (Applied Biosystems, Vilnius, Lithuania) following the manufacturer's protocol. Excess dyes were removed by Performa DTR Gel Filtration Cartridges (Edge Bio, San Jose, CA, USA), and then sequences were run on a 3,500 Genetic Analyzer (Applied Biosystems, Vilnius, Lithuania). Electropherograms were analysed using Sequencing analysis and Sequence Scanner software packages. Consensus sequences for *katG* and *rpoB* were obtained by ClustalW analysis, deposited in GenBank (accession numbers MW821664 and MW821663, respectively), and compared with the corresponding sequence of the reference strain *M. tuberculosis H37Rv (*GenBank accession number AL123456.3).

## Results

In May 2019, during routine inspections, an adult non-neutered medium-size crossbreed male dead dog was found by the Local Health Authority in a gipsy encampment located in the province of Benevento, Southern Italy, and presented to the Istituto Zooprofilattico Sperimentale del Mezzogiorno (IZSM) of Portici, Naples, Italy, in order to investigate the cause of death, with the suspicion of abuse. No anamnestic data were available. The dog was submitted to a complete necropsy. On the basis of the decomposition of the carcass, which showed bloating, skin slippage, and presence of fly larvae, the time elapsed since death was estimated around 15 days (22).

**Figure 1:**
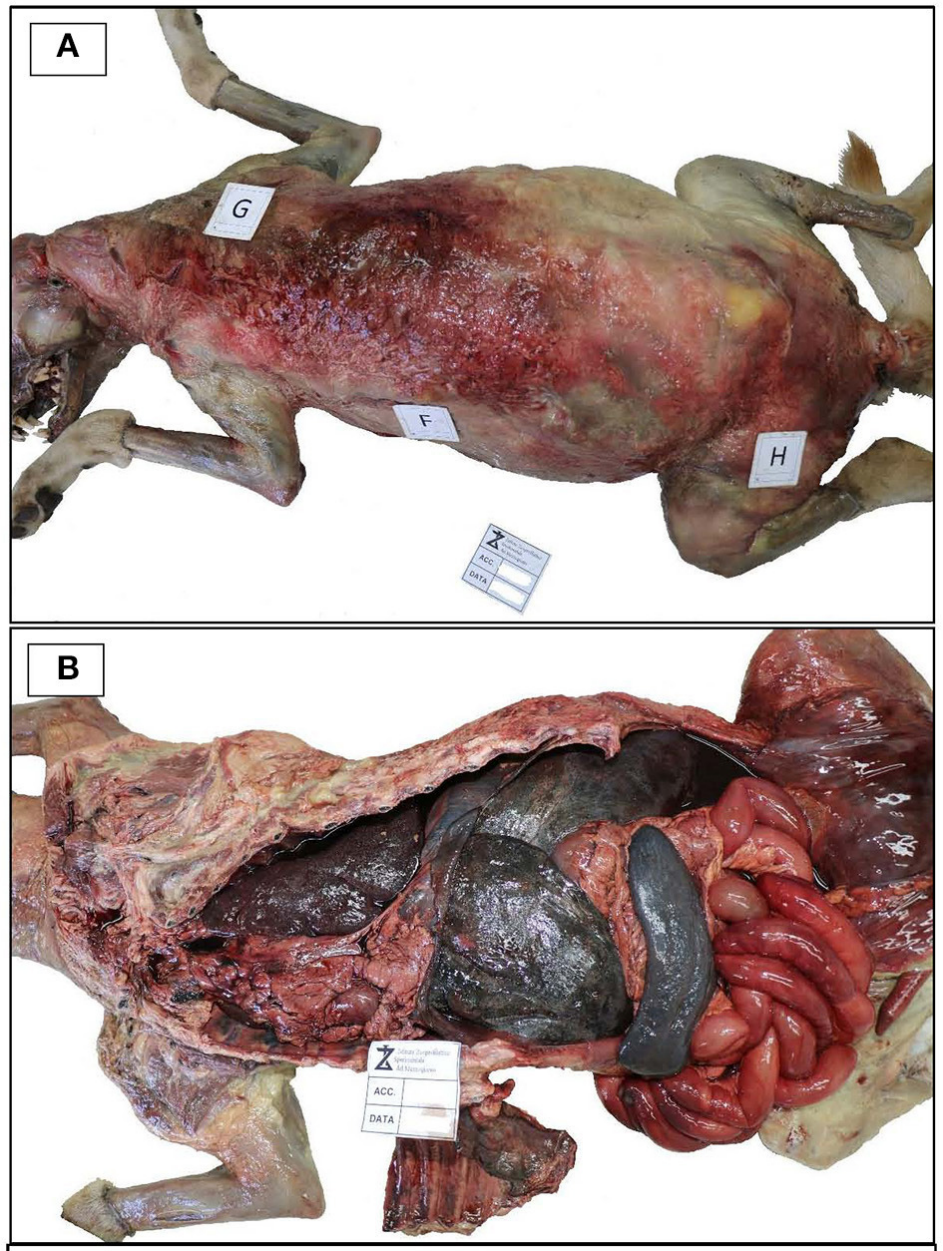
Necropsy examination. **(A)** Necropsy examination showing diffused hematomas on the dorsal regions of the skimmed body. **(B)** Body cavity effusions, hepatosplenomegaly and hemorrhagic enteritis.

**Figure 2:**
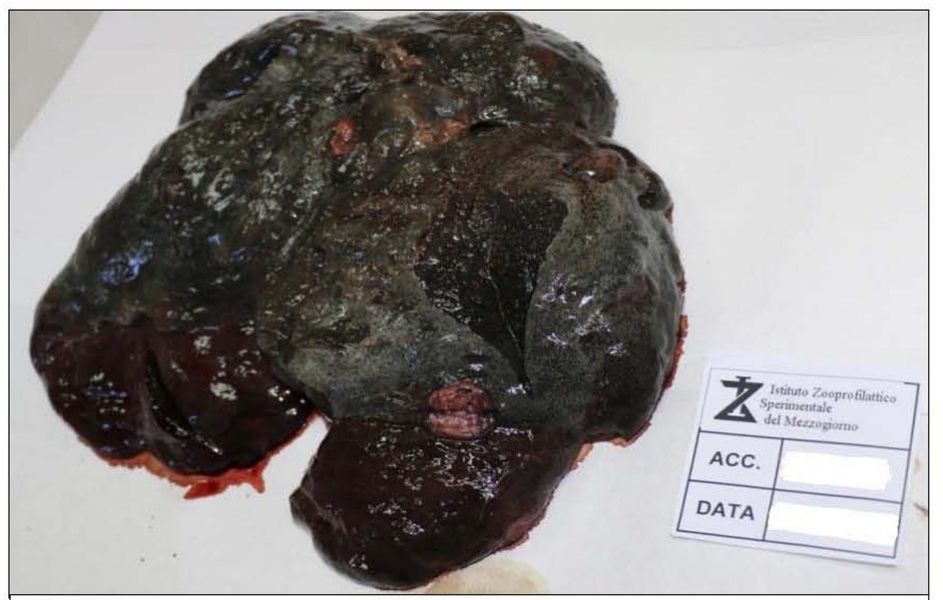
Liver granulomatous lesions. Multifocal granulomatous hepatic lesions.

External examination showed a good state of nutrition, with a body condition score of 7/9; explorable lymph nodes were normal, and mucous membranes were not valuable due to the decomposition stage. The dog was skinned for the evaluation of subcutis and muscles. Diffused hematomas were observed on ventral and dorsal regions (Figure 1A), while in the coxal region, an old bullet wound was found. After opening, body cavities showed postmortem meteorism and colliquation of the viscera. Gross necropsy findings included severe bloody abdominal, pleural, and pericardial effusions, moderate enlargement of liver and spleen, left adrenal gland neoplasm, hemorrhagic enteritis with enlargement of mesenteric lymph nodes (Figure 1B), and multifocal firm greyish-red circumstanced lesions (0.5–1 cm) with central mineralization on the diaphragmatic surface of the liver (Figure 2). The colliquation status of the carcass made impossible to observe other macroscopic lesions in the other organs. On the basis of these findings, the suspicion of cruelty to the animal was confirmed.

**Figure 3:**
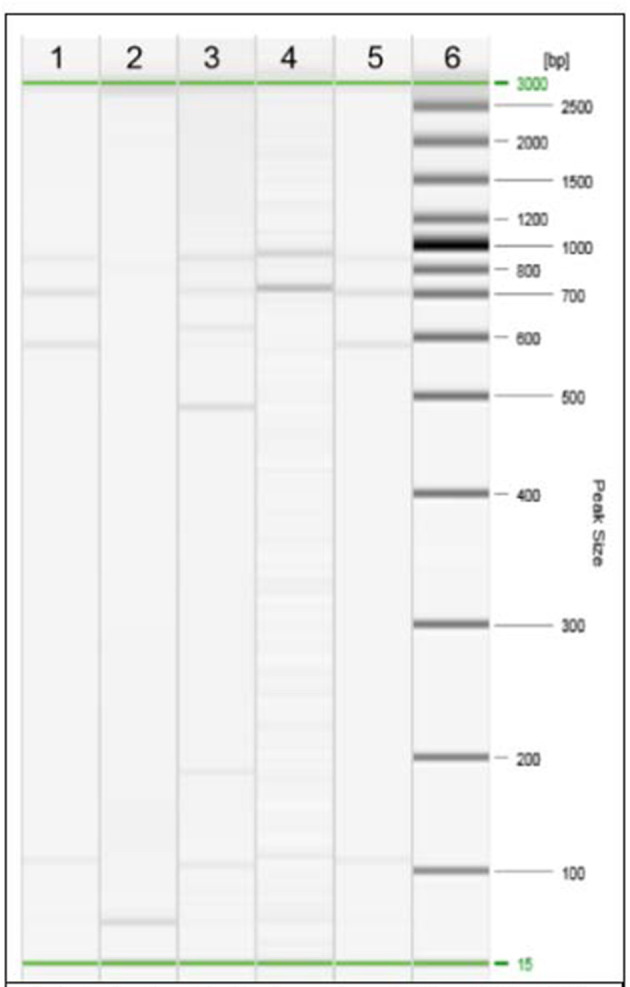
Restriction Fragment Length Polymorphism (RFLP) analysis of the isolated strain of Mycobacterium. Line 1, strain under study; line 2, negative control; line 3, *M. bovis*; line 4, *M. microti*; line 5, *M. tuberculosis*; line 6, DNA ladder.

Specimens from liver lesions were submitted to histopathological examination. The decomposition stage of the tissues made possible the visualisation on histologic sections of nodular formations only, with focuses of mineralization. Bacterial culture was performed according to the procedures described by the World Organisation for Animal Health in the Manual of Diagnostic Tests and Vaccines for Terrestrial Animals (19). Suspected Mycobacterial colonies were grown and then subjected to Ziehl–Neelsen staining that revealed acid-fast bacilli. The isolated colonies were submitted to molecular analyses. First, a real-time PCR for the detection of *Mycobacterium tuberculosis Complex* was conducted, and then species identification was performed using gyrB-restriction fragment length polymorphism (gyrB-RFLP) analysis, which indicated that the fragment sizes were compatible with *M. tuberculosis* (Figure 3). Next, the specimens were submitted to Mycobacterial interspersed repetitive units (MIRUs)–variable number tandem repeat (VNTR) that showed that the isolate could be classified as Spoligotyping International Type 42 (SIT42). The presence of rifampin and isoniazid resistance was evaluated by amplification and sequencing of the *rpoB* and *katG* genes. The isolated strain displayed no mutations in the considered genes, likely suggesting susceptibility to first-line TB drugs.

The Local Health Authority notified the owners the implications of the findings and informed them on the related potential zoonotic risk. In that occasion, a second adult male dog, which lived in contact with the positive one, was found in the same encampment. The authorities submitted both the dog and the owners to the Manteaux test. The owners were uncooperative; thus, we were not able to know the results of their skin test, while unresponsive results were obtained for the dog. For public health safety reasons, the second dog was destined to euthanasia. Next, the carcass was presented to the Unit of Forensic Veterinary Medicine of the IZSM for necropsy and microbiological examinations. No gross lesion was observed, and negative results were obtained by bacteriological analysis.

## Discussion

*Mycobacterium tuberculosis* is recognised as the main species causing human tuberculosis. Although the pathogen was considered exclusively human-related, reports in domestic and wild animals are recently increasing (23).

In dogs, TB is rarely reported and the disease is considered an anthropozoonosis, since humans are believed to represent the main source of the pathogen for companion animals (3, 4, 12) after prolonged aerosol exposure with active TB people (24). Tuberculosis in companion animals shows mostly a subclinical course. When dogs develop clinical signs, symptoms are often non-specific and vary according to the organs affected (3). The most common primary sites of Mycobacterial infection are lungs and pulmonary lymph nodes (3, 16, 25) for the high oxygen tension required by Mycobacteria for survival and replication (25). Nevertheless, different localizations have been reported, such as disseminated, cardiac, and intra-abdominal lesions (16, 25, 26). In this report, multifocal mineralized hepatic lesions (diameter of 0.5–1 cm), hemorrhagic enteritis with enlargement of mesenteric lymph nodes, peritoneal fluid, and intra-thoracic signs of pleural and pericardial effusions were observed, showing an extensive abdominal involvement.

In both humans and in dogs, hepatic micronodular tuberculomas with a diameter of 1–3 cm are commonly described as a consequence of secondary spread through the hematogenous route of primary pulmonary tuberculosis (25, 27), although, as described by Engelmann and colleagues in a case of intra-abdominal canine TB, pulmonary tract findings can be inconspicuous (25). Ascitis and pleural and pericardial effusions are often observed in human and canine *Mtb* infections, which characterise the extrapulmonary TB “wet type,” and the fluids may or may not contain the bacilli (28–30).

The zoonotic role of infected dogs in the transmission of *M. tuberculosis* to humans is still unclear (25). Some authors reported that dogs, despite a disseminated TB and a close contact with humans, did not infect their owners (16, 26). Nevertheless, accidental infection of veterinarian personnel during necropsy has been described (26). Thus, dog-to-human transmission can be considered uncommon (15). In our case, due to the poor compliance of the owners, their health status and the source of the infection for the index dog remain unclear. Another critical point is the role of dogs in the transmission of *Mtb* to other animals. TB dogs can shed the pathogen through nasal discharge, urine, and faeces, so that they can be responsible for environmental contamination for other animals (13). These findings are supported by the evidence of Bonovska et al. (14) who observed the development of lung tuberculosis in a control dog during canine experimental infection. Likewise, in another study, a dog-to-cat transmission was suspected (26). In our study, we did not observe either organ lesions at necropsy or bacteriological growth of *M. tuberculosis* in the healthy dog living in the same habitat with the infected one, even though we had no information about the time of their contact. Moreover, primary hepatic tuberculosis is rare, and the liver does not represent a favourable environment for the growth of TB bacilli for low oxygen tension (31); indeed, it is associated to low transmission rates. For this reason, it is likely that a dog-to-dog transmission could be considered uncommon.

Our results indicate that the isolated *Mtb* strain is the SIT42 type, which is among the most widespread spoligotype in humans worldwide (31–34), thus supporting the hypothesis of a human-to-dog transmission. This genotype is also reported to be strongly associated to multidrug resistance (MDR), mostly among human immunodeficiency virus (HIV) coinfected patients (35, 36). Antibiotic resistance represents one of the main challenges in the treatment of TB and has become a major difficulty for the global control of the disease (37, 38). Rifampin and isoniazid are first-line anti-TB agents. Relevant mutations in the beta subunit of the RNA polymerase (*rpoB*) gene and mutations in the catalase-peroxidase enzyme gene (*katG*) can be involved in RMP and INH resistance to *M. tuberculosis*, respectively (37). Nevertheless, in the present study, we observed no mutation on the *rpoB* and *katG* genes of our SIT42 isolate, suggesting susceptibility to both rifampin and isoniazid relative antibiotics.

In conclusion, canine tuberculosis is rarely described, and, in many cases, companion animals can probably remain misdiagnosed or undetected (16). In our study, a dog that underwent necropsy for suspicion of cruelty to the animal led to the accidental recovery of *M. tuberculosis* SIT42, thus supporting the hypothesis of underestimation of the disease in this species. Moreover, our findings have a particular concern in public health, as dogs could act a role of a reservoir for the pathogen with implications in occupational health (26).

## Data Availability Statement

The original contributions presented in the study are included in the article/supplementary material, further inquiries can be directed to the corresponding author/s.

## Ethics Statement

Ethical review and approval was not required for the animal study because The Istituto Zooprofilattico Sperimentale is the official laboratory designed by the Italian Ministry of Health. According to National regulation and internal policy, ethical approval was deemed unnecessary.

## Author Contributions

LV, LC, GB, and ES drafted the manuscript. GG, GM, and GF conceded the revised the study. PC, AC, MR, and RS conducted necropsy, microbiological, and biotechnological exams. All authors contributed to the article and approved the submitted version.

## Conflict of Interest

The authors declare that the research was conducted in the absence of any commercial or financial relationships that could be construed as a potential conflict of interest.
